# Retinal Changes in an ATP-Induced Model of Retinal Degeneration

**DOI:** 10.3389/fnana.2016.00046

**Published:** 2016-04-29

**Authors:** Felix P. Aplin, Kirstan A. Vessey, Chi D. Luu, Robyn H. Guymer, Robert K. Shepherd, Erica L. Fletcher

**Affiliations:** ^1^Centre for Eye Research Australia, Royal Victorian Eye and Ear Hospital, East MelbourneMelbourne, VIC, Australia; ^2^Department of Anatomy and Neuroscience, The University of MelbourneMelbourne, VIC, Australia; ^3^The Bionics Institute, East MelbourneMelbourne, VIC, Australia; ^4^Department of Surgery (Ophthalmology), The University of MelbourneParkville, VIC, Australia; ^5^Medical Bionics Department, The University of MelbourneMelbourne, VIC, Australia

**Keywords:** retina, gliosis, animal models, adenosine triphosphate, retinal degeneration, feline, photoreceptor cells, retinitis pigmentosa

## Abstract

In rodents and felines, intravitreal administration of adenosine triphosphate (ATP) has been shown to induce photoreceptor death providing a tractable model of retinal degeneration in these species. This study investigated the long term effects of photoreceptor loss in an ATP induced feline model of retinal degeneration. Six normal sighted felines were unilaterally blinded using intravitreal ATP injections and assessed using electroretinography (ERG) and optical coherence tomography (OCT). At 30 h (*n* = 3) or 12 weeks (*n* = 3) post-injection, the animals were euthanized and the eyes enucleated. Retinae were sectioned and labeled using immunohistochemistry for markers of cell death, neural remodeling and gliosis. Ongoing cell death and retinal degeneration was observed in the outer retina at both 30 h and 12 weeks following unilateral ATP injection. Markers of mid to late-stage retinal remodeling such as cell displacement and aberrant neurite growth were observed in the inner retina at 12 weeks post-injection. Ganglion cells appeared to remain intact in ATP injected eyes. Müller cell gliosis was observed throughout the inner and outer retina, in some parts completely enveloping and/or displacing the surviving neural tissue. Our data suggests that the ATP injected feline retina continues to undergo progressive retinal degeneration and exhibits abnormalities consistent with a description of retinal remodeling commonly seen in other models of retinal degeneration. These findings validate the use of intravitreal ATP injection in feline as a large animal model of retinal degeneration which may aid in development of therapies aiming to restore visual function after photoreceptor degeneration.

## Introduction

Retinitis pigmentosa (RP) is an inherited retinal dystrophy characterized by dysfunction and progressive loss of rod and/or cone photoreceptors in the retina. In the advanced stages of disease, the inner retina undergoes remodeling and cell death as a secondary reaction to the initial disease etiology (Milam et al., 1998; Strettoi et al., 2002; Jones et al., 2003; Marc et al., 2003; Sullivan et al., 2003; Kalloniatis and Fletcher, 2004). Understanding the mechanisms behind the secondary inner retinal disease processes in these conditions is vital as we move towards for the restoration of functional vision in patients with these conditions using retinal prostheses.

Animal models of photoreceptor degeneration allow researchers to explore the pathological processes involved in these diseases. The need for safe, rapid and effective large eyed animal models of retinal disease has intensified as we move to an era of restorative strategies such as neural prostheses, optogenetics and photoreceptor sheet transplantation (Dahlmann-Noor et al., 2010; O’Brien et al., 2012; Seiler and Aramant, 2012; Shepherd et al., 2013; Singh et al., 2013; Zrenner, 2013) as well as stem cell and gene replacement therapies (Limb and Daniels, 2008; Dahlmann-Noor et al., 2010). Large-eyed animal models of blindness such as the P347L rabbit and the P23H pig models of retinal degeneration (Jones et al., 2011; Sommer et al., 2011) allow for proof of concept trials and the assessment of efficacy for these strategies before they are translated into the human. In order to maximize the potential for restoration of vision in disease of photoreceptors, a large animal model must recapitulate the degenerative process observed in humans, including and importantly the inner retinal neurons, especially the ganglion cells, which must remain intact and capable of passing information to higher centers. Even after successful clinical application of a new technology, animal models remain relevant as they enable researchers to better understand the impact of primary and secondary degenerative processes on optimal device or treatment performance (Bertschinger et al., 2008).

We previously developed a feline model of retinal degeneration using unilateral adenosine triphosphate (ATP) injection, which lead to rapid photoreceptor cell death followed by progressive degeneration over a 12 week period (Aplin et al., 2014). However, the remodeling events in the ATP-injected feline, have not yet been characterized. We define retinal remodeling as degenerative changes occurring secondary to a primary retinal insult that conform to the description of remodeling events as originally described by Jones et al. (2003, 2005) and Marc et al. (2003) seen commonly throughout retinal degenerations. Whilst a thorough characterization of retinal remodeling has been described in ATP injected rat (Puthussery and Fletcher, 2009; Vessey et al., 2014), it is important to understand the remodeling processes involved in the ATP-injected feline model of retinal degeneration as there may be species specific differences that are important when using the models for research into vision restorative therapies. The feline eye is larger, contains a higher cone photoreceptor density compared to the rat and has an area of central higher acuity vision (area centralis) which may affect the profile of degeneration (Steinberg et al., 1973). Furthermore, ATP-induced phototoxicity is thought to be mediated by purinergic receptors, especially the receptor P2X7, and there may be differences in retinal purine expression between species (Puthussery and Fletcher, 2009; Vessey et al., 2012). We therefore aimed to histologically characterize the extent of retinal remodeling and degenerative processes in a chronic, unilateral, ATP-induced feline model of retinal degeneration.

## Materials and Methods

### Anesthesia and Intraocular Injection of ATP

Normally sighted adult laboratory felines (*n* = 6) were used in this study. Three animals were a subset of a larger cohort used at the completion of a previous study (Aplin et al., 2014). Treatment of animals complied with the Association for Research in Vision and Ophthalmology Statement for Use of Animals in Ophthalmic and Vision Research, and the National Health and Medical Research Council’s (NHMRC) “Australian Code of Practice for the Care and Use of Animals for Scientific Purposes” (2013) and the “Prevention of Cruelty to Animals Act” (1986; and amendments). The study was approved by the Royal Victorian Eye and Ear Hospital Animal Ethics Committee (RVEEH AEC; #12/256AB).

Unilateral retinal degeneration was induced in the animals using an intravitreal injection of ATP. This procedure has been described at length elsewhere (Puthussery and Fletcher, 2009; Aplin et al., 2014; Vessey et al., 2014). In brief, Animals were anesthetized with a subcutaneous injection of ketamine (20 mg/kg, Ilium Ketamil, Troy Laboratories, NSW, Australia) and xylazine (2 mg/kg, Ilium Xylazil-20, Troy Laboratories, NSW, Australia) and a 100 μL solution of sterile phosphate buffered saline (PBS, 0.9%) containing 0.2M adenosine tri-phosphate hydrate (Sigma Pharmaceuticals, VIC, Australia) and 0.2 mg dexamethasone (4 mg/ml, Dexamethasone, Aspen Australia, NSW, Australia) was injected into one eye. The fellow eye received a sham injection of 0.2 mg dexamethasone in 100 μL PBS as a control.

### Structural and Functional Assessments

Twin-flash electroretinography (ERG) and optical coherence tomography (OCT) were used to assess the effect of ATP injection on retinal structure and function pre-operatively and at 12 weeks post-injection. All assessment and analysis methodologies performed on these animals have been described in previous studies (Aplin et al., 2014). In short, the integrity of the surviving retinal structure was assessed with OCT scans across the area centralis (Spectralis; HRA + OCT, Heidelberg Engineering, Heidelberg, Germany) and quantified using a custom script in ImageJ (version 1.48^1^, provided in the public domain by the National Institutes of Health, Bethesda, MD, USA). Visual function was assessed using full field, twin-flash ERG (Espion; Diagnosys LLC, Lowell, MA, USA) which allowed for a separation of rod- and cone-pathway mediated function.

### Tissue Collection and Histology

At 30 h (*n* = 3) or 12 weeks (*n* = 3) after ATP injection, animals were anesthetized using ketamine (20 mg/kg) and xylazine (2 mg/kg) and then euthanized with an overdose of sodium pentobarbitone (150 mg/kg, Troy Laboratories, intracardiac, NSW, Australia). Both eyes were enucleated and the eye anterior to the ciliary body removed. The remaining eyecups were fixed in 4% paraformaldehyde for 30 min and washed in phosphate buffered solution (PB, 0.9%). Sections were equilibrated in graded sucrose solutions (10%, 20%, 30% w/v in PB) for 30 min each and stored overnight. The control and ATP-injected retinae were embedded in optimal cutting temperature compound (Tissue-Tek, CA, USA), frozen with liquid nitrogen and cut into serial sections at 12 μM on a cryostat (Microm, Walldorf, Germany). Sections were collected on Poly-L-lysine coated slides (Menzel-Glaser, Braunschweig, Germany) and stored at −20°C.

Cell death was measured using a commercially available fluorometric terminal dUTP nick-end labeling (TUNEL) kit as per Manufacturer’s instructions (DeadEnd Fluorometric TUNEL system, TB235; Promega, Madison, WI, USA). Fluorescence immunohistochemistry was used to assess retinal cell survival, morphology and markers of retinal remodeling near the area centralis. Immunohistochemical techniques followed methodology previously described in ATP injected rat and feline (Aplin et al., 2014; Vessey et al., 2014). After sectioning, slides were defrosted and washed in PB for 10 min. Sections were incubated at room temperature overnight in primary antibody (detailed below) diluted in antibody buffer (3% normal goat serum (NGS), 1% bovine serum albumin (BSA), 0.5% Triton-X in PB). Primary antibody solution was removed with PB washes and the sections incubated at room temperature for 3 h in antibody buffer containing secondary antibody diluted 1:500 (goat anti-mouse AlexaFluor 488, goat anti-rabbit AlexaFluor 594, or goat anti-guinea pig Alexa-Fluor 643; Life Sciences, VIC, Australia) and a nuclear stain diluted 1:300 (40, 6-diamidino-2-phenylin-dole (DAPI); Life Sciences, VIC, Australia). Finally sections were rinsed in PB and coverslipped with a Mowiol-based antifade mounting medium (Polysciences, Inc., PA, USA). Photomicrographs were taken on a LSM 5 Meta laser scanning confocal microscope (Carl Zeiss AG, Gottingen, Germany) with a 20× air or 40× oil objective. A linear contrast stretch was applied post-acquisition in all images to enhance contrast. A minimum of four images were taken for each vertical section, and three sections were imaged per eye. Of these 36 sections, two were chosen for display: one to best represent the average level of degeneration across all images and the other to best represent the more acutely affected regions of the degenerated retina.

Primary antibodies were selected to examine possible remodeling events at 12 weeks post ATP injection in both the inner and outer retina. In order to examine photoreceptor survival and remodeling we labeled rod photoreceptor outer segments with mouse Rho 4D2 (R4D2; 1:50; Abcam, CA, USA; Fariss et al., 1997), cone photoreceptor outer segments with red/green and blue rabbit cone opsin (1:500; Millipore, VIC, Australia; Linberg et al., 2001) and the cone photoreceptor inner and outer segments with fluorescent labeled peanut agglutinin (PNA; 1:200; Vector Laboratories, CA, USA; Linberg et al., 2001). In order to examine synaptic and bipolar cell changes in the rod pathway we labeled retinae with mouse C-terminal-binding protein 2 (RIBEYE; 1:1000; BD Biosciences, NSW, AUS) or rabbit anti-Protein Kinase Cα (PKCα; 1:500; Sigma-Aldrich, NSW, Australia) and guinea pig anti-Vesicular Glutamate Transporter (VGLUT1; 1:1000; Millipore, VIC, Australia). PKCα is a label for rod bipolar cells (Greferath et al., 1990; Lewis et al., 1998). RIBEYE labels ribbon synapses in the inner and outer plexiform layers (IPL/OPL), and some inner retinal cell bodies (Fisher et al., 2005; Lewis et al., 2005). VGLUT1 labels glutamatergic synaptic terminals (Fyk-Kolodziej et al., 2004). In order to examine the effect of ATP-induced retinal degeneration on amacrine and horizontal cells we labeled retinae with mouse Calbindin (1:500; Swant, Bellinzona, Switzerland) and VGLUT1 (1:1000; Millipore, VIC, Australia) or mouse Calretinin (1:500; Swant, Bellinzona, Switzerland) and VGLUT1 (1:1000; Millipore, VIC, Australia). Calbindin primarily labels horizontal cells and also some ganglion cells and amacrine cells (Pasteels et al., 1990; Lewis et al., 1998). Calretinin primarily labels amacrine cells and some ganglion cells (Pasteels et al., 1990; Macneil et al., 2009). Ganglion cells were labeled with anti-RNA-binding protein with multiple splicing (RBPMS). RBPMS is a reliable label of retinal ganglion cell bodies in mouse, rat, guinea pig, rabbit and monkey retina (Rodriguez et al., 2014). A preliminary analysis confirmed an identical pattern of labeling in the feline.

Müller cell and astrocyte morphology was labeled using rabbit antiglial fibrillary acid protein (GFAP; 1:10,000; Dako, CA, USA) and mouse anti-Glutamine synthetase (GS; 1:1000; Millipore, VIC, Australia). GFAP labels astrocytes and gliotic Müller cells in the retina (Lewis et al., 1988; Vessey et al., 2011; Vessey and Fletcher, 2012). GS labels normal functioning or gliotic Müller cells with GS expression (Lewis et al., 1988) To examine the effect of ATP-induced retinal degeneration on microglia we labeled retinae with rabbit polyclonal anti-ionized calcium-binding adaptor molecule 1 (IBA-1; 1:1000; Wako Pure Chemical Industries, VA, USA). IBA-1 exclusively labels microglia in the feline central nervous system and retina (Ahmed et al., 2007; Zhang et al., 2011). The number of photoreceptor nuclei in the outer nuclear layer (ONL), the thickness of the ONL and RBMPS-labeled retinal ganglion cell counts were quantified per 100 μm of retina across the area centralis manually using ImageJ. Photoreceptor nuclei density was calculated by multiplying the cell count in each 100 μm bin by the total area of the ONL in that bin.

### Statistical Analysis

Analyses were performed on ERG amplitudes, OCT measurements of retinal thicknesses, ONL thickness and photoreceptor nuclei density using Statistical Software (Graphpad Prism v.4, Graphpad Software, CA, USA; SigmaPlot v12.5, Systat Software, CA, USA). ERG, OCT and histological quantifications were analyzed using a non-parametric Wilcoxon signed rank test (Wilcoxon), compared to the baseline for OCT and ERG measurements and to the fellow sham injected eye for histological quantification. The in-text results and all graph error bars represent one standard deviation from the mean (SD). Results were considered significant when *p* < 0.05.

## Results

### ATP Induced Retinal Degeneration Causes Cell Death and Reorganization of the Outer Retina

We first examined the level of functional and structural loss present in this cohort of animals at 12 weeks post ATP injection using OCT and ERG analysis. This data is a subset of a larger population used in the previous study (Aplin et al., 2014). At 12 weeks post ATP injection, there was a significant reduction in average rod and cone a-wave amplitudes compared to the pre-injection baseline values (rod a-wave reduction = 84.97% ± 11.45%; Wilcoxon; *p* < 0.001; cone a-wave reduction = 75.85% ± 16.15%; Wilcoxon; *p* < 0.023; *n* = 3 animals). There was no measurable reduction in a-wave amplitudes in the sham-injected control eyes. There was also a significant reduction in total retinal thickness and ONL thickness at 12 weeks post ATP injection compared to baseline in all animals (total retinal thickness reduction = 34.69% ± 15.76%; Wilcoxon; *p =* 0.036; ONL thickness reduction = 75.20% ± 13.11%; Wilcoxon; *p* < 0.001; *n* = 3 animals). There was no measurable reduction in retinal thicknesses in the sham injected eyes compared to baseline.

**Figure 1:**
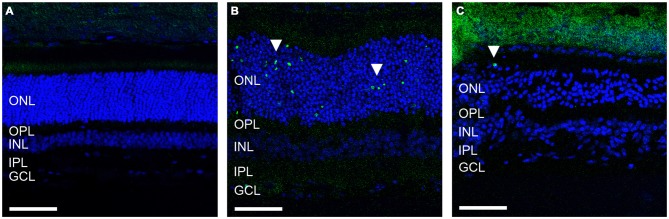
**ATP injection leads to secondary progressive loss of photoreceptors. (A–C)** Representative sections of control and ATP-injected retinae labeled for cell death (TUNEL; green) and cell nuclei (DAPI; blue) in a control **(A)**, 30 h after injection **(B)** and 12 weeks after intravitreal injection with ATP **(C)**. White arrows indicate a TUNEL positive nuclei. ONL, outer nuclear layer; OPL, outer plexiform layer; INL, inner nuclear layer; IPL, inner plexiform layer; GCL, ganglion cell layer. Scale bars = 100 μM.

Given the ongoing loss of ERG waveform reported previously in the ATP-injected feline, we sought to determine whether retinal cell death was still occurring 12 weeks post ATP injection in these animals and how this compared to acute cell death only 30 h post-injection in an identical injection concentration. Figure 1 shows representative vertical sections of feline retinae in a control (Figure 1A), at 30 h (Figure 1B) and at 12 weeks (Figure 1C) post ATP injection, labeled for cell nuclei (DAPI; blue) and for cell death (TUNEL; green). Many TUNEL positive cells can be seen in the 30 h condition, showing an acute loss of photoreceptors in response to ATP injection (white arrows). There were still some TUNEL positive nuclei 12 weeks post-injection (white arrow); however, TUNEL positive cells were qualitatively fewer in number than in the 30 h condition. This suggests that the majority of photoreceptor loss occurs as an acute response to ATP injection, but that progressive photoreceptor cell death may continue to occur up to 12 weeks after the initial insult.

**Figure 2:**
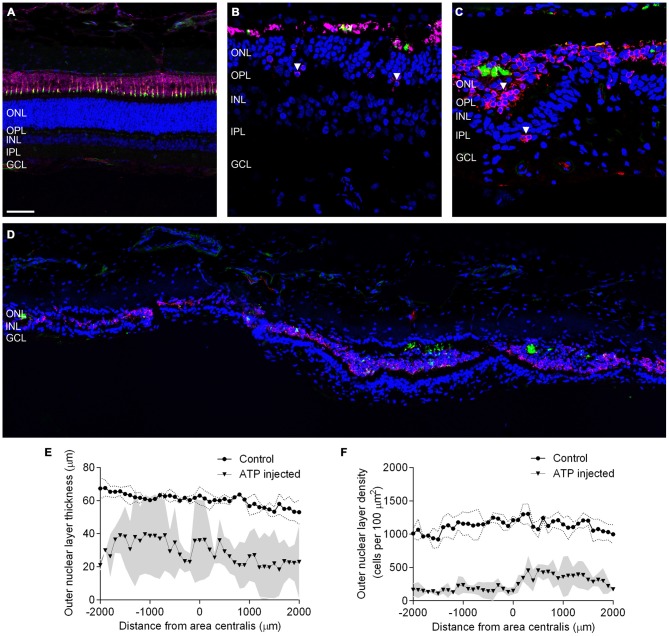
**Surviving rod and cone photoreceptors undergo regionally variable degeneration. (A–D)** Representative sections of control **(A)** and ATP-injected retinae at 12 weeks post injection **(B,C)** labeled for rhodopsin (R4D2; pink) cone opsins (cone opsin; red), cone outer segments (PNA; green) and cell nuclei (DAPI; blue). **(D)** Representative tile scan of ATP injected retinae at 12 weeks post injection labeled for rhodopsin (R4D2; pink) cone opsins (cone opsin; red), cone outer segments (PNA; green) and cell nuclei (DAPI; blue). White arrows indicate abnormal rhodopsin and cone opsin expression. **(E,F)** Average ONL thickness **(E)** and density **(F)** as a function of distance from the center of the area centralis in control and ATP injected eyes. The gray area corresponds to one standard deviation (SD) from the mean in the ATP injected eyes, while the dotted line corresponds to one SD from the mean in control eyes. ONL, outer nuclear layer; OPL, outer plexiform layer; INL, inner nuclear layer; IPL, inner plexiform layer; GCL, ganglion cell layer. Scale bars = 100 μM.

The presence of photoreceptor nuclei alone does not indicate that residual photoreceptors remain capable of phototransduction. To better explore the overall integrity of the surviving photoreceptors we examined markers for photoreceptor opsins and outer segments across the area centralis. Figures 2A–D shows representative vertical sections of feline retinae labeled for cell nuclei (DAPI; blue), Rhodopsin (R4D2; pink), Cone opsins (Cone opsin; red) and cone outer segments (PNA; green) in a control (Figure 2A) and at a higher magnification in 12 week (Figures 2B,C) ATP injected eyes. White arrows indicate abnormal rhodopsin and cone opsin expression located in the photoreceptor cell bodies. Figure 2D shows a representative tile scan vertical section of feline retinae across the area centralis at 12 weeks post injection, labeled for photoreceptor opsins and outer segments as above. By 12 weeks the outer retina had become highly disorganized, with a few relatively normal areas interspersed between areas of little to no normal outer segment survival. In the worst affected areas, photoreceptor opsin was either absent or primarily expressed in cell bodies, with some evidence of abnormal cell displacement to other retinal layers (Figure 2C, white arrow). In order to examine variations in photoreceptor loss across the area centralis in the ATP-injected eyes we then quantified ONL thickness (Figure 2E) and density of photoreceptor nuclei (Figure 2F) in 100 μm sections across the retina. There was a significant reduction in the thickness of ATP-injected retinae compared to the same region in fellow eye controls (Sham injected mean = 60.83 μm ± 2.871; ATP injected mean = 28.89 μm ± 12.53; Mean reduction = 52.51% ± 18.48; Wilcoxon; *p =* 0.042; *n* = 3 animals). Density was also significantly reduced compared to fellow eye controls (Sham injected mean = 1117 cells/100 μm^2^ ± 35.37; ATP injected mean = 257 cells/100 μm^2^ ± 44.79; Mean reduction = 77.10% ± 6.403; Wilcoxon; *p* < 0.001; *n* = 3 animals). The average thickness of the ONL in ATP injected eyes varied considerably across the measured area (Figure 2E). In contrast, photoreceptor density remained uniformly low even in thicker areas (Figure 2F).

### ATP Induced Retinal Degeneration Remodels the Inner Retina by 12 Weeks Post Injection

**Figure 3:**
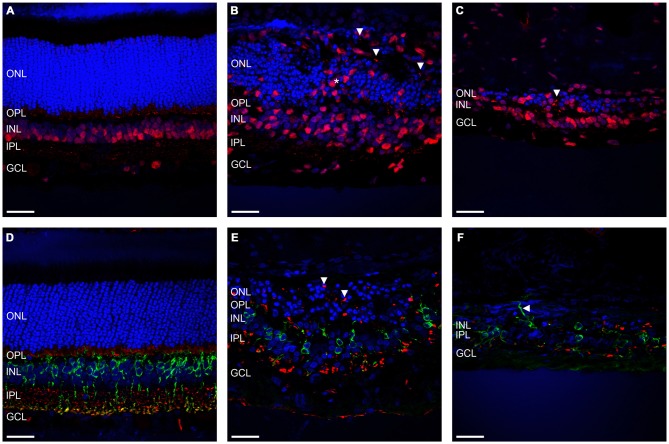
**Rod bipolar cells and synapses show evidence of retinal remodeling at 12 weeks after ATP injection. (A–C)** Representative sections of control **(A)** and ATP-injected retinae at 12 weeks post injection **(B,C)** labeled for ribbon synapses and inner retinal cell bodies (RIBEYE; red) and cell nuclei (DAPI; blue). **(D–F)** Representative sections of control **(D)** and ATP-injected retinae at 12 weeks post injection **(E,F)** labeled for glutamatergic synapses (VGLUT1; red), rod bipolar cells (PKCα; green), and cell nuclei (DAPI; blue). Vertical white arrows indicate displaced synapses. The horizontal arrow in **(F)** indicates a displaced cell process. The asterisk indicates a column of displaced inner retinal neurons migrating into the ONL. ONL, outer nuclear layer; OPL, outer plexiform layer; INL, inner nuclear layer; IPL, inner plexiform layer; GCL, ganglion cell layer. Scale bars = 100 μM.

Given that severe loss and remodeling of the ONL would be expected to lead to remodeling of inner retinal processes, we examined the effect of ATP-induced chronic photoreceptor degeneration on bipolar cells and synapses in the inner nuclear layer (INL) and OPL/IPL. Figures 3A–C shows representative vertical sections of feline retinae labeled for cell nuclei (DAPI; blue) and ribbon synapses/inner retinal nuclei (RIBEYE; red) in a control (Figure 3A) and two ATP injected eyes at 12 weeks post injection (Figures 3B,C). Figures 3D–F shows representative vertical sections of feline retinae labeled for cell nuclei (DAPI; blue), synaptic terminals (VGLUT1; red) and rod bipolar cells (PKCα; green) in a control (Figure 3D) and two ATP injected eyes at 12 weeks post injections (Figures 3E,F). White arrows in all images show evidence for displaced synaptic terminal expression or displacement of INL neuronal processes. Asterisk in Figure 3B indicates a column of substantial inner retinal neuronal displacement into the ONL. Sections for ATP injected eyes were chosen in order to represent both the average level of degeneration (Figures 3B,E) and the full extent of degeneration across the retina (Figures 3C,F). We found that RIBEYE consistently labeled a population of inner retinal neuron somas in the cat, as has been previously reported (Fisher et al., 2005). This labeling revealed the movement of inner retinal neurons into the outer retina towards the RPE (Figure 3B). Bipolar cells in the INL were still present even in severely degenerated areas, although there was evidence of late stage remodeling as evidenced by the displaced cell bodies, synaptic terminals and cell processes. The integrity of the OPL appeared highly compromised, with little to no VGLUT or RIBEYE expression in degenerated areas and displaced punctate labeling of VGLUT and RIBEYE extending as far as the RPE.

**Figure 4:**
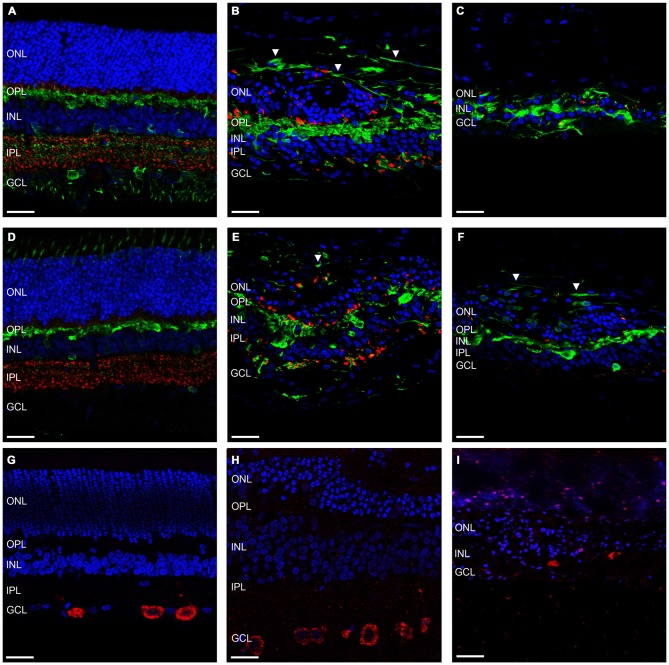
**Amacrine and ganglion cells show evidence of retinal remodeling at 12 weeks after ATP injection. (A–C)** Representative sections of control **(A)** and ATP-injected retinae at 12 weeks post injection **(B,C)** labeled for glutamatergic synapses (VGLUT1; red), a subset of horizontal, amacrine and ganglion cells (calretinin; green), and cell nuclei (DAPI; blue). **(D–F)** Representative sections of control **(D)** and ATP-injected retinae at 12 weeks post injection **(E,F)** labeled for glutamatergic synapses (VGLUT1; red), a subset of horizontal, amacrine and ganglion cells (calbindin; green), and cell nuclei (DAPI; blue). **(G–I)** Representative sections of control **(G)** and ATP-injected retinae at 12 weeks post injection **(H,I)** labeled for retinal ganglion cells (RBPMS; red) and cell nuclei (DAPI; blue). White arrows indicate evidence of aberrant neurite bundle formation in the outer retina. ONL, outer nuclear layer; OPL, outer plexiform layer; INL, inner nuclear layer; IPL, inner plexiform layer; GCL, ganglion cell layer. Scale bars = 100 μM.

Further to this, we then examined the effect of ATP induced photoreceptor degeneration on horizontal cell, amacrine cell and ganglion cell morphology. Figures 4A–C shows representative vertical sections of feline retinae labeled for cell nuclei (DAPI; blue), synaptic terminals (VGLUT1; red) and amacrine, ganglion cell and horizontal cells (Calretinin; green) in a control (Figure 4A) and two ATP injected eyes at 12 weeks post injection (Figures 4B,C). Figures 4D–F shows representative vertical sections of feline retinae labeled for cell nuclei (DAPI; blue), synaptic terminals (VGLUT1; red) and a different subset of amacrine, ganglion cell and horizontal cells (Calbindin; green) in a control (Figure 4D) and two ATP injected eyes at 12 weeks post injection (Figures 4E,F). Figures 4G–I shows representative vertical sections of feline retinae labeled for cell nuclei (DAPI; blue) and ganglion cell bodies (RBPMS; green) in a control (Figure 4G) and at 12 weeks post injection (Figures 4H,I). White arrows in all images show evidence for abnormal neurite outgrowth into the outer retina. Sections were chosen in order to represent the both the average level of degeneration (Figures 4B,E,H) and the full extent of degeneration across the retina (Figures 4C,F,I). Amacrine cell populations appeared to remain relatively well conserved in ATP injected eyes, although amacrine and/or horizontal cell processes could be seen to form remodeled neurite bundles in the outer retina. The IPL was disorganized with a reduction in the expression of synaptic terminals. There was no significant reduction in ganglion cell count compared to controls (control = 16.56 ± 5.232 cells/100 μm; ATP injected = 12.52 ± 5.668 cells/100 μm; Wilcoxon; *p* = 0.430; *n* = 3 animals). The ganglion cell layer (GCL) appeared mostly intact 12 weeks post-injection of ATP, although qualitatively there was clearly some cell loss in the worst affected areas, as well as ganglion cell displacement into the IPL (Figure 4I).

### ATP Induced Retinal Degeneration Causes Pathological Changes in Non-neural Retinal Tissue

**Figure 5:**
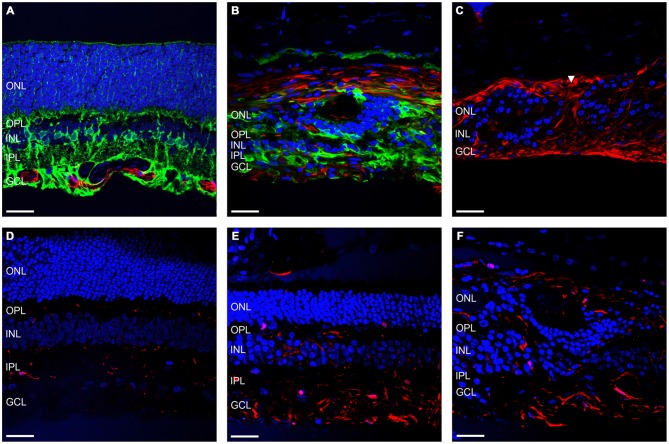
**Müller cells and microglia alter their expression in response to ATP injection. (A–C)** Representative sections of control **(A)** and ATP-injected retinae at 12 weeks post injection **(B,C)** labeled for astrocytes and gliotic Müller cells (GFAP; red), Müller cells (GS; green; **A,B** only), and cell nuclei (DAPI; blue). **(D–F)** Representative sections of control **(D)** and ATP-injected retinae at 12 weeks post injection **(E,F)** labeled for microglia (IBA-1; red) and cell nuclei (DAPI; blue). White arrow indicates location of a glial column extending throughout the retina. ONL, outer nuclear layer; OPL, outer plexiform layer; INL, inner nuclear layer; IPL, inner plexiform layer; GCL, ganglion cell layer. Scale bars = 100 μM.

Another important facet of retinal degenerations are the morphological changes in the glial components of the retina. Figures 5A,B shows representative vertical sections of feline retina labeled for cell nuclei (DAPI; blue), astrocytes and/or gliotic Müller cells (GFAP; red) and normal functioning Müller cells (GS; green) in a control (Figure 5A) and an ATP injected eye at 12 weeks post injection (Figure 5B). Figure 5C shows representative sections of retina 12 weeks after ATP injection labeled for DAPI (blue) and GFAP (red) only, to better display specific gliotic remodeling events. Figures 5D–F shows representative sections of retina labeled for cell nuclei (DAPI; blue) and microglia (IBA-1; red) in a control (Figure 5D) and 12 weeks post injection (Figures 5E,F). The white arrow in Figure 5C indicates the location of a glial column that has displaced normal retinal layers. It was evident that Müller cells in the retina had undergone severe gliosis in response to ATP-induced retinal degeneration. In many parts of the retina, gliotic Müller cells had completely entombed the outer retina via the formation of a glial scar, at times with a thickness comparable to the total surviving neural retina (Figures 5B,C). Occasionally, glial columns infiltrated across all retinal layers, creating areas devoid of neural tissue (Figure 5C). Microglial activity was increased in ATP-injected eyes at 12 weeks post injection (Figures 5E,F), especially in the inner retina but also within the outer retina in more degenerated regions (Figure 5F).

.

## Discussion

This study characterized long term changes in retinal morphology induced by intravitreal ATP injection in a feline model of retinal degeneration. Photoreceptor loss was associated with abnormalities in the inner and outer neural retina and widespread Müller cell gliosis. The markers for retinal remodeling described in our study are consistent with findings in the ATP injected rat (Vessey et al., 2014) and other models of photoreceptor degeneration (Jones et al., 2005; Marc et al., 2008). Our data suggest that ATP-induced retinal degeneration in the feline follows a similar progression of late-stage retinal remodeling to other models of photoreceptor degeneration, albeit at a much more rapid rate compared to inherited retinal degeneration in the feline (Narfström, 1985; Narfström and Nilsson, 1987).

### ATP Injection Causes Ongoing Photoreceptor Degeneration

Photoreceptor cell death occurred acutely at 30 h post injection, with evidence of slower photoreceptor death continuing out to 12 weeks post injection. ATP is normally hydrolyzed rapidly into adenosine within hours of entering the body (Zhang et al., 2006; Köles et al., 2011), so the mechanism by which photoreceptor cells continue to die at 12 weeks cannot be a direct effect of the ATP injection. Chronic photoreceptor degeneration may thus indicate a secondary photoreceptor loss in these animals, possibly by mechanisms independent from the initial disease etiology. Secondary cell loss is common to photoreceptor degenerations but does not yet have a complete explanation in the literature (Strettoi et al., 2002; Jones et al., 2003, 2005; Marc et al., 2003; Fletcher, 2010).

The inner and outer segments did not consistently express rod or cone opsins by 12 weeks after ATP injection. Instead, cone and especially rhodopsin expression appeared to have primarily migrated into the cell body of the photoreceptors. A similar pattern of opsin expression has been reported in other models of retinal degeneration (Gao et al., 2002; Fletcher et al., 2011) and is likely to indicate photoreceptor stress and dysfunction, and may directly contribute to ongoing photoreceptor cell death (Alfinito and Townes-Anderson, 2002). We had previously reported a smaller reduction in ONL thickness using OCT compared to ERG a-wave amplitude loss (Aplin et al., 2014). Our currents results imply that this may be due to survival of non-functional photoreceptor cell bodies which contribute to the appearance of a thicker ONL but do not contribute to light—evoked responses using ERG. Furthermore, although the thickness of the ONL varied considerably between animals and across the retina in ATP injected animals, the density of photoreceptor nuclei was uniformly low across the retina. Our results suggest that, at least in rapid-acting models of photoreceptor degeneration, it is not possible to assess retinal survival by solely quantifying the thickness of the ONL, as this may give an over-inflated estimation of the number of functional photoreceptors still present within the retina. As ONL thickness was seen to reduce over time in our previous study (Aplin et al., 2014), we might expect if this model was taken out to longer time points these dysfunctional photoreceptors would be gradually cleared and the thickness of the ONL would better correlate to residual ERG a-wave amplitude as it does in some human retinal degenerations (Cideciyan et al., 2008). A study of Leber’s Congenital Amaurosis in human patients showed regions of thickened retina at the beginning stages of the disease progression in some patients (Jacobson et al., 2007), which was noted as likely due to an increase in gliosis within the tissue from acute cell death. We may not see this effect in slower forms of retinal degeneration such as RP simply because these conditions take many years to progress in the human, giving time for the glial tissue and photoreceptor debris to clear. Thus, a superficially thick retinal profile may naturally result from any form of rapid photoreceptor death and is not solely a feature of ATP induced phototoxicity.

### ATP Injection Causes Inner Retinal Neural Remodeling and Widespread Müller Cell Gliosis

A common feature of many photoreceptor degenerations is a progressive and pathological remodeling of the neural retina in response to a primary photoreceptor loss (Marc et al., 2003; Jones et al., 2005). In this feline model of ATP-induced retinal degeneration, we observed many markers of mid to late-stage retinal remodeling present in both the INL and GCLs of the inner retina. Specifically, there was patchy loss of bipolar cells, gross displacement of INL neurons into the outer and inner nuclear and synaptic layers, a generalized loss of normal markers of synaptic function, evidence for abnormal horizontal or amacrine cell neurite growth into the outer retina and the presence of non-neural (gliotic) tissue especially within the outer retina and GCLs. The severity of inner retinal remodeling appeared to be relatively inconsistent across the retina, with the most severe remodeling occurring in regions devoid of even residual photoreceptor nuclei. This is consistent with both human degenerations and animal models of blindness that characteristically show non-uniform cell survival and remodeling, with neural survival particularly associated with the survival of cone photoreceptors (Jones et al., 2003, 2005). The ganglion cell population was conserved according to cell counts; however the worst affected areas had an obvious visual reduction in the number of surviving RGCs so we believe it likely that there is some reduction that our low sample size could not detect statistically. Retinal ganglion cell morphology is relatively well maintained in inherited photoreceptor degenerations (Strettoi et al., 2002; Jones et al., 2005; Mazzoni et al., 2008) although there are reports of up to 70% RGC loss in the most severely degenerated human cases (Santos et al., 1997). Some level of ganglion cell survival is vital for the success of several visual restoration strategies including retinal prosthesis and photoreceptor sheet transplants (Seiler and Aramant, 2012; Zrenner, 2013). Thus, these findings help to validate the use of the ATP-injected model of retinal degeneration to trial these types of restoration strategies.

GFAP-positive Müller cell gliosis occurred as a result of ATP injection, with a thick glial scar commonly observed across the inner and outer retina, entombing the surviving neural tissue. Müller cell gliosis was present in all areas of the retina but appeared thickest in degenerating and degenerated regions. In many areas, the lateral glial scar and/or vertical glial columns formed by Müller cells appeared to completely displace the surrounding neural tissue. Müller cell gliosis appeared to be even more severe and disruptive in our specific model of retinal degeneration in the feline compared to other models (Jones et al., 2003, 2011; Vessey et al., 2014). Although it is not clear why gliosis appears to be so prevalent in our model we propose that this is likely a combination of direct activation of glial cells in the retina by ATP injection (Köles et al., 2011), the focal retinal detachments we observed in these animals (Aplin et al., 2014) and the rapid rate of primary photoreceptor degeneration leading to an activation of the gliotic response. The observed increased microglia activity suggested an ongoing inflammatory stimulus in these animals.

Overall our results closely mimic a previous study which characterized retinal remodeling events in ATP-injected rat retinae (Vessey et al., 2014). Retinal remodeling is often separated into three distinct stages. The initial evidence of photoreceptor cell death is referred to as phase 1 retinal remodeling; gross outer retina and subtle inner retinal changes indicate phase 2 remodeling; and total loss of photoreceptors and gross inner retinal changes define phase 3 remodeling (Marc et al., 2003; Jones et al., 2005). Our data suggest that at 12 weeks post ATP injection, the feline retina is undergoing general phase 2 remodeling with localized phase 3 remodeling in areas with total photoreceptor loss, and very occasional phase 1 remodeling occurring in areas with greater than normal photoreceptor survival. Again, this time course remains very similar to that reported previously in the ATP-injected rat model of retinal degeneration (Vessey et al., 2014).

The mechanism behind ATP mediated photoreceptor cell death is still not completely understood. ATP-mediated cell death is thought to play a role in inherited retinal degenerations (Puthussery and Fletcher, 2009; Fletcher, 2010), and although the process likely involves direct phototoxic activation of the P2X7 purinergic receptor (Puthussery and Fletcher, 2004; Puthussery et al., 2006; Vessey et al., 2012, 2014), purinergic receptors are also found throughout the retina on neural, glial and endothelial cells (Puthussery and Fletcher, 2004; Puthussery et al., 2006; Mitchell and Reigada, 2008). Thus, a secondary effect on photoreceptors or other neural cells must also play some part in the disease phenotype observed in the ATP injected feline and rodent retina. For example, purinergic receptors are found on cells within the retinal pigment epithelium (Tovell and Sanderson, 2008; Wan et al., 2011), and death or dysfunction of RPE cells could also lead to photoreceptor loss as is observed in other models of retinal degeneration such as sodium iodate (NaIO_3_) phototoxicity (Enzmann et al., 2006). Purinergic receptors are also found on glial cells within the central nervous system (CNS) and retina, and play a role in neural-glial cell signaling (Fields and Burnstock, 2006; Newman, 2006). It is possible that the increased gliotic activity observed in our model may be in part a primary effect of high extracellular ATP rather than a secondary response to photoreceptor degeneration. An increased gliotic response could contribute to the acute and ongoing loss of photoreceptors and potentially inner retinal neurons in the ATP induced model of photoreceptor degeneration (Bringmann et al., 2009). Further to this it is possible that ATP has direct pathological interactions with other cells types in the retina which were otherwise not revealed from our analysis, especially as retinal purines have not been fully characterized in the feline. However, given how closely our findings resemble previous descriptions of retinal remodeling including the ATP injected and P23H rat, and the wide array of remodeling markers identified in our tissue, we do not think our data can be explained as a purely primary effect of ATP phototoxicity.

The ATP injected model of retinal degeneration provides a novel model of photoreceptor degeneration with unique set of advantages and disadvantages compared to other transgenic and toxic models of retinal degeneration in the feline and other larger-eyed animal models. The ATP induced model of retinal degeneration in the feline bypasses many of the limitations of comparable transgenic models such as the Abyssinian cat (Narfström and Nilsson, 1987; Kang Derwent et al., 2006) or rhodopsin transgenic pig (Petters et al., 1997; Li et al., 1998), as it is cheap, rapid, widely available and the injection protocol is relatively simple to perform. However, ATP does not closely mimic the initial disease etiology or rate of disease progression in human RP, which typically occurs over a period of many years. Fast-acting phototoxic compounds as models of retinal degeneration are thus a compromise between accessibility and the specificity of the model, and are more suitable for therapeutic interventions that are in themselves relatively generic such as visual prostheses or photoreceptor transplants.

The ATP model of retinal degeneration also benefits from being a unilateral model of retinal degeneration. Other common toxic models of photoreceptor degeneration such as N-methyl-N-nitrosourea (MNU), NaIO_3_ and iodoacetic acid (IAA; Kiuchi et al., 2002; Noel et al., 2012; Chen et al., 2014) have similar disease progression timelines but must be applied systemically and cause bilateral blindness. Although there have been some recent progress in translating MNU into a unilateral model (Rösch et al., 2014), the compound is highly carcinogenic and the long term-effects of unilateral MNU injection still remain uncharacterized. A unilateral model of blindness is ideal as it allows for a fellow eye control. In a larger eyed model, unilateral blindness also reduces housing and support costs for the animals and alleviates the ethical concerns involved with bilateral blindness in a primarily visual animal.

In conclusion, we assessed neural and glial morphology using immunohistochemistry in feline retinae at 30 h and 12 weeks after unilateral intravitreal injection with ATP as a toxic model of photoreceptor degenerations. There was evidence of severe photoreceptor loss at 30 h and progressive photoreceptor loss and outer retinal degeneration 12 weeks post ATP injection. Markers in inner retinal neurons corresponding to phase 2 and phase 3 retinal remodeling were present throughout ATP injected retinae. All retinal layers showed evidence of disorganization and neural loss, although ganglion cells remained relatively well conserved. Extensive and severe Müller cell gliosis was observed throughout the retina, particularly in areas with greater retinal degeneration. In the worst affected areas, fibrotic tissue had completely displaced the neural retina. Our data suggest that the ATP-induced model of retinal degeneration in the feline follows a pattern of secondary neural and glial change that closely mimics remodeling a seen in other models of retinal degeneration. This characterization should provide a valuable reference tool for future studies using the ATP injected model of photoreceptor degeneration to assess the viability of visual restorative therapies.

## Author Contributions

FPA designed, performed and analyzed experiments, and wrote the manuscript. KAV designed and analyzed experiments and edited the manuscript. CDL, RHG, RKS supervised and designed experiments and edited the manuscript. ELF supervised, designed and analyzed experiments, and edited the manuscript.

## Acknowlegments

This work was supported by the Australian Research Council (ARC) through its Special Research Initiative (SRI) in Bionic Vision Science and Technology grant to Bionic Vision Australia (BVA) and by the National Health and Medical Research Counci (NHMRC) project grant (APP1021042; APP1061419) to ELF by Retina Australia. The Bionics Institute and CERA receive Operational Infrastructure Support from the Victorian Government.

## Conflict of Interest Statement

The authors declare that the research was conducted in the absence of any commercial or financial relationships that could be construed as a potential conflict of interest.

## Footnotes

^1^http://imagej.nih.gov/ij/
